# Interview Administration of PROMIS Depression and Anxiety Short Forms

**DOI:** 10.3928/24748307-20190626-01

**Published:** 2019-09-06

**Authors:** Bayley J. Taple, James W. Griffith, Michael S. Wolf

## Abstract

**Background:**

Health literacy reflects a person's reading and numeracy abilities applied to understanding health-related information. These skills may influence how patients report symptoms, leading to underestimates or overestimates of symptom severity. No prior studies have examined health literacy measurement bias.

**Objective:**

The purpose of the current study was to determine whether PROMIS (Patient-Reported Outcomes Measurement Information System) anxiety and depression short forms, administered by interview, capture symptoms equally across health literacy groups. We examined the psychometric properties of PROMIS anxiety and depression short forms using differential item functioning (DIF) analysis by level of health literacy.

**Methods:**

The sample analyzed included 888 adults, age 55 to 74 years, in Chicago, IL. Health literacy was measured using the Test of Functional Health Literacy in Adults. PROMIS short forms assessed anxiety and depression.

**Key Results:**

DIF was present in 3 of 8 depression items, and 3 of 7 anxiety items. All items flagged for DIF had lower item-slopes for people with limited health literacy.

**Conclusions:**

Items with DIF were less strongly related to anxiety and depression, and thus less precise. Overall, impact of DIF on PROMIS scores was negligible, likely mitigated by interview administration. Although overall test impact of health literacy was minimal, DIF analyses flagged items that were potentially too complex for people with limited health literacy. Design and validation of patient-reported surveys should incorporate respondents with a range of health literacy and methods to identify and reduce measurement bias. **[*HLRP: Health Literacy Research and Practice*. 2019;3(3):e196–e204.]**

**Plain Language Summary:**

This study suggests that people with limited health literacy may respond differently to questions about depression and anxiety than people with adequate health literacy. Therefore, it is important to be aware of differences in literacy ability when creating and using questionnaires.

Health care considers self-report measures a vital sign (Dahl, Saeger, Stein, & Huss, 2000; Glasgow, Kaplan, Ockene, Fisher, & Emmons, 2012). Patients may not accurately comprehend questions about their health if limited health literacy interferes with understanding. Moreover, patients with low health literacy may feel uncomfortable answering questions about their health. Interview administration may mitigate the effects of limited health literacy on self-report. Some research has used a method where trained interviewers read self-report questions to patients and record patient responses, but whether this method is effective is unknown. Therefore, we analyzed this method of interview administration.

This study examined how health literacy influences interview assessment of anxiety and depression. Mood and anxiety disorders are a public health concern (Kessler et al., 2003; Kessler, Petukhova, Sampson, Zaslavsky, & Wittchen, 2012). The World Health Organization predicts that depression will be the second highest cause of disability by 2020 (Kessler & Bromet, 2013). The lifetime prevalence of unipolar depression and anxiety disorders have been reported at 17% and 29%, respectively (Kessler et al., 2005). Accurate assessment of depression and anxiety is necessary. As such, the Patient-Reported Outcomes Measurement Information System (PROMIS) includes depression and anxiety measures (Pilkonis et al., 2011), but it is unknown whether these items are precise across levels of health literacy. The current study examines how health literacy affects the psychometric properties of PROMIS depression and anxiety items (Cella et al., 2007, 2010, 2015; Pilkonis et al., 2011).

Health literacy is the “degree to which individuals have the capacity to obtain, process, and understand basic health information and services needed to make appropriate health decisions” (Kindig, Panzer, Nielsen-Bohlman, 2004; Ratzan & Parker, 2000). People with limited health literacy are, on average, less knowledgeable about their disease and treatment, less able to successfully perform self-care tasks, and have poorer self-efficacy. This translates to worse health outcomes including greater hospitalization, mortality risk, use of emergency services, and decreased use of preventive care (e.g., Baker, 2006; Baker, Parker, Williams, & Clark, 1998; Berkman, Sheridan, Donahue, Halpern, & Crotty, 2011; Kindig, Panzer, Nielsen-Bohlman, 2004; Paasche-Orlow & Wolf, 2007; Vandenbosch et al., 2016).

People with limited health literacy have difficulty with information that is needed to care for oneself and navigate the health care system (Wolf et al., 2012). Thus, providers must find ways of communicating clearly with patients across levels of health literacy. Health literacy is distinct from literacy or educational attainment alone; it is specific to communication within the health care context (Nutbeam, 2008). Research has yet to explore differential item functioning (DIF) by health literacy. DIF is a way to statistically quantify whether group membership, in the current study having limited versus adequate health literacy, affects the relationship that a questionnaire item has to the concept it measures (depression or anxiety in this case). Jones and Gallo (2002) found DIF by educational attainment on the Mini-Mental State Examination; cognitive errors were overestimated in participants with lower education. Teresi et al. (2009), found DIF by education in 1 of 32 PROMIS depression items. Health literacy is determined by many sociodemographic variables, including education, but health literacy is a stronger predictor of health outcomes than education alone (Paasche-Orlow & Wolf, 2007). Therefore, health literacy has implications for health care utilization. Research is needed on improved means of assessment for people with limited health literacy.

## Objective

The purpose of the present study was to analyze the psychometric properties of PROMIS Short Form v1.0 Depression - 8b and PROMIS Short Form v1.0 - Anxiety - 7a by level of health literacy. We hypothesized that DIF across level of health literacy would be observed for items on both short forms. If DIF was present, we examined whether it was uniform or non-uniform. We calculated correlations between depression, anxiety, and health literacy. We expected our sample to reflect the well-known association between depression and anxiety. To our knowledge, this is the first study to examine the impact of health literacy on DIF.

## Methods

The present study is a secondary analysis of “LitCog,” a National Institute on Aging study (for further details, see Serper et al., 2014; Smith, Curtis, Wardle, Wagner, & Wolf, 2013; Wolf et al., 2012). LitCog assessed health literacy and cognition in older adults. The sample included 888 English-speaking patients, age 55 to 74 years, recruited from an academic general internal medicine clinic and four federally qualified health centers in Chicago, IL.

## Procedure

Participants completed two structured interviews 7 to 10 days apart. A trained research assistant guided patients through a series of assessments that, on Day 1, included basic demographic information, socioeconomic status, comorbidity, health literacy, PROMIS measures, and an assessment of performance on everyday health tasks. On Day 2, the same patients completed a cognitive battery to measure processing speed, working memory, inductive reasoning, long-term memory, prospective memory, and verbal ability. Northwestern University's Institutional Review Board approved the study. All measures used in the present analyses were administered on Day 1.

## Measures

### Depression and Anxiety

The National Institutes of Health conducted an initiative to design standardized questionnaires to evaluate patient-reported outcomes with reliable scores and valid interpretations (Cella et al., 2007, 2010; Fries, Bruce, & Cella, 2005; Hays, Bjorner, Revicki, Spritzer, & Cella, 2009; Liu et al., 2010). We examined the psychometric properties of PROMIS anxiety and depression short forms, administered by interview, across levels of health literacy. PROMIS Short Form v1.0 Depression - 8b comprises eight items rated on 5-point frequency scales (*never* to *always*). All items begin with “In the past seven days…” Example depression items included “I felt hopeless” and “I felt I had nothing to look forward to” (Pilkonis et al., 2011). PROMIS Short Form v1.0 - Anxiety - 7a includes seven items rated on 5-point frequency scales. Example anxiety items included “I felt nervous” and “I found it hard to focus on anything other than my anxiety.” Raw total scores for both scales were transformed into T scores. The United States population mean T score is, by definition, 50 with standard deviation of 10. Both surveys show excellent internal consistency as demonstrated by the mean adjusted item–total correlations (depression: *r* = .83; anxiety: *r* = .79) and alpha coefficients (depression: α = .95; anxiety: α = .93; Pilkonis et al., 2011). In LitCog, an interviewer verbally administered the depression and anxiety short forms.

### Health Literacy

Health literacy was measured using the Test of Functional Health Literacy in Adults (TOFHLA; Parker, Baker, Williams, & Nurss, 1995), one of the most common health literacy assessments (Baker, 2006). The numeracy section is interview based; participants receive prompts, and the interviewer poses a set of questions and records responses. Numeracy items test comprehension of directions for health tasks (e.g., taking medication as prescribed and keeping medical appointments). The literacy portion is paper based; participants work independently to answer multiple choice questions assessing comprehension. Literacy content includes health care documents (e.g., sections of an informed consent form and a Medicaid application; Parker et al., 1995). Total scores range from 0 to 100, with numeracy and literacy subscales each ranging from 0 to 50. A total score greater than or equal to 75 indicates adequate health literacy, whereas less than 75 reflects limited health literacy. The TOFHLA has high internal consistency (Cronbach's alpha = 0.98) and test-retest reliability (Spearman-Brown coefficient = 0.92). Moreover, it demonstrates excellent construct validity as it is highly correlated with measures of literacy—the Rapid Estimate of Adult Literacy in Medicine (Spearman's rho = 0.84) and the Wide Range Achievement Test-Revised (Spearman's rho = 0.74; Parker et al., 1995).

### Differential Item Functioning

DIF can be used as a method to identify whether level of health literacy impacts the relationship between a questionnaire and the concept it measures (i.e., depression or anxiety in this study). DIF exists when “an item favor[s] matched examinees from one group over another” (Shealy & Stout, 1993). Thus, respondents from different groups consistently answer items differently (Reeve & Fayers, 2005). DIF exists when there is a difference in the strength of the relationship between a questionnaire item and a concept across groups. DIF is derived from Item Response Theory (IRT; for review see Cho, Suh, & Lee, 2016). If an item displays DIF, the level of depression or anxiety may be under- or overestimated in people with limited health literacy. DIF can be uniform or non-uniform (Swaminathan & Rogers, 1990). Uniform DIF could exist if the effect of health literacy is constant across levels of depression and anxiety. Non-uniform DIF could be present if, as depression and anxiety change, the effect of health literacy also changes. Even if the level of DIF for individual items is small, if several test items display DIF then the discrepancy between groups may exist for the test as a whole (Shealy & Stout, 1993). This introduces a problem for the construct validity of item score interpretations across groups. Therefore, examining DIF across groups with distinct health literacy capabilities for PROMIS anxiety and depression scales is crucial for accurate assessment. DIF analysis may flag items that may be difficult for people with limited health literacy and may be a useful adjunct to qualitative reviews of items and cognitive interviewing (Collins, 2014; Miller, Chepp, Willson, & Padilla, 2014) during test development.

## Analytical Strategy

We performed DIF analyses on each item of the PROMIS short forms for anxiety and depression, using the *lordif* package in R (Choi, Gibbons, & Crane, 2011). A graded response model is an appropriate IRT model because item responses were polytomous and ordered (Reeve & Fayers, 2005; Samejima, 2016). Note, *lordif* flags items with DIF based on effect-size measures rather than statistical significance. After conducting DIF analyses, we inspected individual items to determine whether item attributes (e.g., item length) were related to DIF across health literacy levels.

Monte Carlo simulations were used to determine the appropriate cutoff for detecting DIF. The Monte Carlo procedure creates simulated datasets under the null hypothesis of no DIF, creating an empirical distribution of effect sizes (Choi et al., 2011). We selected 500 iterations as sufficient to consistently detect the same items flagged for DIF. An effect size is selected at the tail of the distribution based on a chosen alpha level (Choi et al., 2011). We chose α = .01 to indicate possible DIF and used the McFadden pseudo R^2^ as the effect size.

## Results

### Descriptive Statistics

Descriptive statistics are presented in **Table 1**. Unsurprisingly based on prior work (e.g., Berkman et al., 2011; Paasche-Orlow & Wolf, 2007), level of education differed by health literacy group, such that 14% of people with adequate health literacy had a high school degree or less, compared to 63% of people with limited health literacy. Furthermore, 39% of participants with adequate health literacy completed a graduate degree, compared to only 6% of participants with limited health literacy. Race also differed by health literacy group. Seventy-six percent of people with limited health literacy were Black/African American and 11% were White. Conversely, 61% of people with adequate health literacy were White and 30% were Black/African American.

The sample (*N* = 888) had a mean TOFHLA score of 76.26 ± 16.29. The numeracy and reading subscale mean scores were 32.78 ± 8.65 and 43.45 ± 9.59, respectively. Approximately one-third (32%) of the sample had limited health literacy. The average PROMIS depression and anxiety T scores were 47.8 ± 9.1 and 53.2 ± 8.9, respectively. Mean levels of depression and anxiety of the sample were near the US population mean (T = 50). Bivariate correlations are presented in **Table 2**. As expected, depression and anxiety were strongly and positively associated (*r* = .73, *p* < .001). Although the relationship between depression and anxiety significantly differed by health literacy group, *z* = 2.19, *p* < .05, the effect sizes were similar (*r* = .78 for limited health literacy, *r* = .71 for adequate health literacy).

### Differential Item Functioning

**Figure 1.**
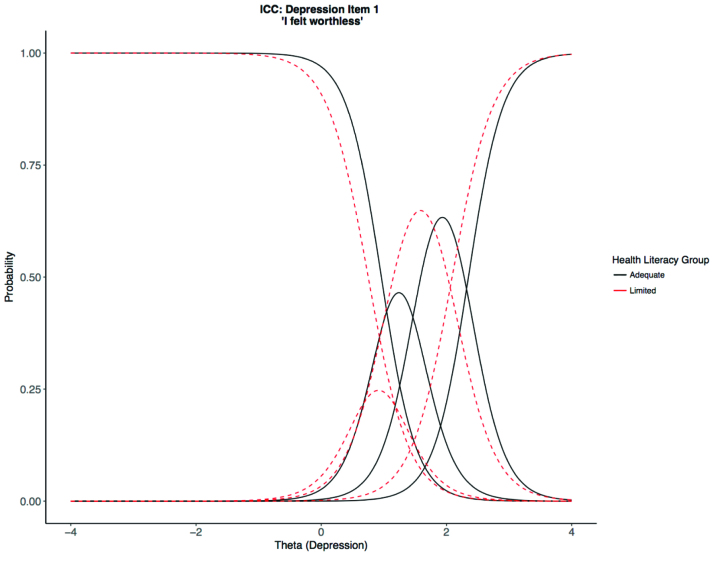
Example item characteristic curves (ICCs) exhibiting differential item functioning for the item “I felt worthless” on depression short form 8b. Theta is the depression metric on a standardized scale (*M* = 0, *SD* = 1 by definition). In this example, there are three rather than four thresholds (curve intersections); if fewer than five participants used a response category, categories were automatically collapsed by *lordif*. In this case, *often* and *always* were collapsed into one category.

DIF analyses in *lordif* allow for one grouping variable in the model; we used health literacy as the grouping variable. Five hundred iterations of Monte Carlo simulation at α = .01 (i.e., the cutoff for the upper 1% of the Monte Carlo distribution) suggested effect sizes that exceeded McFadden pseudo R^2^ = .002 were indicative of possible DIF. Thus, we used R^2^ = .002 as the cutoff for flagging items for DIF in the observed data. A total effect of DIF was present in 3 of the 8 depression items. “I felt worthless” displayed uniform DIF, slope for limited health literacy, a_limited_ = 3.06; slope for adequate health literacy, a_adequate_ = 3.60; R^2^ = .018 (**Figure 1**). “I felt hopeless” displayed uniform DIF, a_limited_ = 4.93; a_adequate_ = 5.61; R^2^ = .007. “I felt depressed” showed non-uniform DIF, a_limited_ = 2.88; a_adequate_ = 4.22; R^2^ = .006.

**Figure 2.**
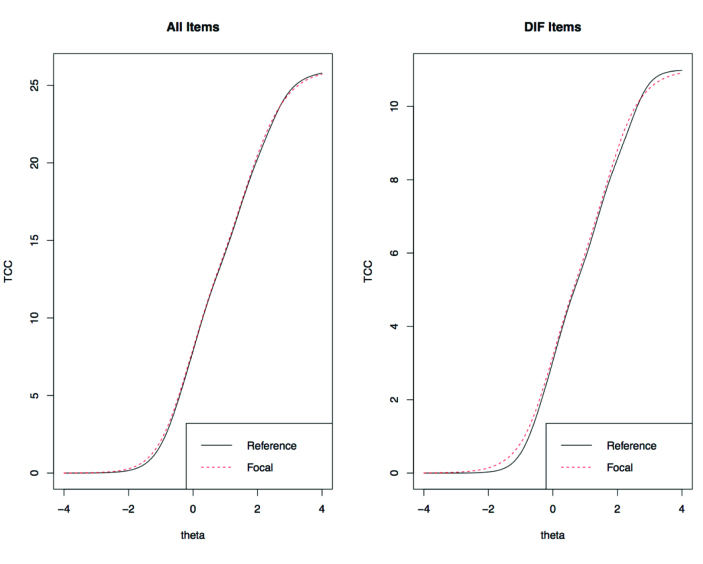

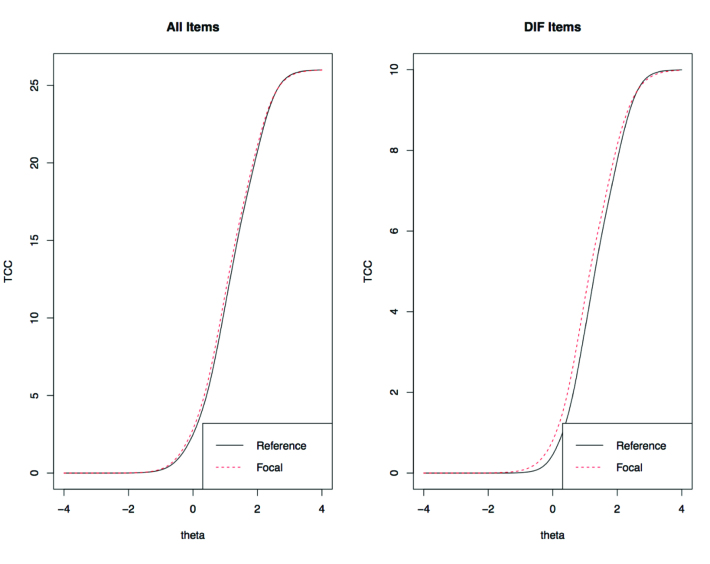
(A) Test characteristic curves (TCCs) for anxiety short form 7a. The TCCs for limited versus adequate health literacy are overlapping, suggesting that measurement precision for the anxiety Patient-Reported Outcomes Measurement Information System (PROMIS) scale is similar across the two groups, across level of anxiety. Reference denotes adequate literacy; Focal denotes limited literacy. Theta is the anxiety metric on a standardized scale (*M* = 0, *SD* = 1 by definition). (B) TCCs for depression short form 8b. The TCCs for limited versus adequate health literacy are overlapping, suggesting that measurement precision for the depression PROMIS scale is similar across the two groups, across level of depression. Reference denotes adequate literacy; focal denotes limited literacy. Theta is the depression metric on a standardized scale (*M* = 0, *SD* = 1 by definition). DIF = differential item functioning.

A small total effect of DIF was present for 3 of the 7 anxiety items. “I felt anxious” had uniform DIF, a_limited_ = 1.91; a_adequate_ = 3.15; R^2^ = .004. “I felt nervous” had uniform DIF, a_limited_ = 3.52; a_adequate_ = 3.92; R^2^ = .004. “I found it hard to focus on anything other than my anxiety” displayed non-uniform DIF, a_limited_ = 1.95; a_adequate_ = 2.78; R^2^ = .004. **Figures 2A–2B** show the test characteristic curves (TCCs) for anxiety and depression, respectively. The overlapping lines suggest that DIF negligibly impacted PROMIS T scores.

## Discussion

As predicted, we observed DIF by health literacy group. Six of 15 items on PROMIS anxiety and depression short forms displayed DIF with small effect sizes (negligible according to the R^2^ < .13 guideline suggested by Choi et al., 2011). TCCs for depression and anxiety scales showed little difference in total response between health literacy groups (**Figures 2A–2B**). Therefore, health literacy did not influence PROMIS T scores administered by interview. Interview administration was employed as a strategy to lessen the impact of health literacy on self-report, because the interviewer can verify participant comprehension (Doak, Doak, Friedell, & Meade, 1998). Even if health literacy did not impact overall test precision for PROMIS short forms, flagging individual items with DIF is valuable because it identifies items that are potentially problematic for people with limited health literacy. As Edelen and Reeve (2007) note—the advantage of IRT applications, including DIF, is “detailed item-level information” (p. 16). If the scales were completed as paper and pencil or on a computer, items may have shown ever greater DIF owing to the need for the participant to read the items.

Other studies have found negligible DIF of PROMIS items yet propose the value of examining DIF as a tool to implement assessments appropriately (e.g., Cook, Bamer, Amtmann, Molton, & Jensen, 2012; Coster et al., 2016; Hong et al., 2016). Moreover, similar effect sizes (pseudo R^2^ = .001 to .003) have been associated with impactful DIF in other studies (Crane et al., 2007). PROMIS was developed through evidence-based design. Hence, these analyses may be informative for questionnaires that have been designed less rigorously; that is, other questions with more complex questions might show even greater DIF. Researchers and clinicians should be cognizant of the effects of health literacy group on assessment. DIF analyses can elucidate issues within individual items. For example, “I felt depressed” may have shown DIF because “depressed” may be a low frequency word in everyday language, is often stigmatized, or it can have different meanings to different people (e.g., sadness vs. loss of interest). The item “I found it hard to focus on anything other than my anxiety” may have exhibited DIF because it requires some metacognition (i.e., thinking about anxiety); it is also longer than other items.

At the item level, health literacy influenced the item characteristic curves of six PROMIS depression and anxiety items, even with interview administration to potentially reduce impact of health literacy. All items flagged for DIF had lower item-slopes for people with limited health literacy (**Figure 1**), suggesting that these items were less strongly related to the constructs of anxiety and depression, and thus, less precise. The current analyses can guide the design of questionnaires to capture patients' symptomatology regardless of health literacy ability. Researchers can calculate an effect size and use the magnitude as a decision-making tool for how to handle each item where DIF is present (Cho et al., 2016). For example, if DIF exceeds the calculated effect size and if item length contributes to DIF, then the item could be revised. The design and validation of questionnaires should incorporate respondents with a range of health literacy and methods to identify and thus eliminate measurement bias. PROMIS measures were developed with reading level in mind, so other questionnaires may exhibit much more DIF if literacy is not considered in the questionnaire-development process.

## Limitations and Future Directions

We acknowledge several limitations. First, the analyses were cross-sectional. Future studies may benefit from examining the longitudinal effect of health literacy on testing. Second, depression and anxiety in this sample were in the average range. Different results might be found in a clinical sample with higher levels of and/or more variability in depression and anxiety. Third, PROMIS was administered in a non-standard way (i.e., by interview). That said, it is possible that health literacy effects would have been greater had the participants been asked to read the questions on their own. Research has shown that people with limited literacy have poorer comprehension of health material, and that methods such as Teach-Back, whereby patients explain their understanding to an interviewer, are recommended (e.g., Kripalani, Bengtzen, Henderson, & Jacobson, 2008). Going forward, it is important to consider methods of improving comprehension, especially in the context of our digital world, where there are disparities in use of technology in people with limited literacy compared to those with adequate literacy (Bailey et al., 2014). Health literacy is a global concept that attempts to integrate various factors (e.g., education, socioeconomic status) that contribute to understanding of health-related information (Paasche-Orlow & Wolf, 2007). Future research may tease apart the individual contributions to health literacy as they relate to depression and anxiety assessment.

## Conclusions

This study addressed the effects of health literacy on DIF of PROMIS anxiety and depression short forms. DIF analysis is important because if questions are not equally applicable (Reeve & Fayers, 2005) across health literacy groups, the validity of interpretations between groups on the measured construct is undermined. Examining individual item characteristics provides rich information about how group disparities impact assessment. Whether or not group differences affect the scale as a whole, placing assessment in the context of the patient informs research and clinical judgment. An item with DIF across health literacy may result in a different testing experience for participants; DIF analysis can identify these items.

## Figures and Tables

**Table 1:** Sample Characteristics

**Characteristic**	**Overall Sample^a^*N* = 900 (%)**	**Adequate Literacy *n* = 604 (%)**	**Limited Literacy *n* = 284 (%)**

Mean age, years (*SD*)	63.1 (5.5)	62.8 (5.3)	63.8 (5.8)

Sex			
Male	618 (69)	416 (69)	194 (68)
Female	282 (31)	188 (31)	90 (32)

Education			
High school or less	264 (29)	83 (14)	179 (63)
Some college or technical school	206 (23)	131 (22)	70 (25)
College graduate	174 (19)	153 (25)	18 (6)
Graduate degree	256 (28)	237 (39)	17(6)

Ethnicity/race			
White	404 (45)	368 (61)	30 (11)
Black/African American	401 (45)	179 (30)	216 (76)
Hispanic/Latinx	21 (2)	10 (2)	11 (4)
Asian	17 (2)	10 (2)	7 (2)
Other	52 (6)	33 (5)	19 (7)
Missing	5 (<1)	4 (<1)	1 (<1)

Note.

a Health literacy groups include 888 participants total; 12 did not complete the Test of Functional Health Literacy in Adults.

**Table 2:** Bivariate Correlations Among the Study Variables

**Variable**	***N***	**1**	**2**	**3**	**4**

1. Anxiety T Score	897				
2. Depression T Score	899	.73^***^			
3. TOFHLA Numeracy Score	892	−.11^***^	−.20^***^		
4. TOFHLA Reading Score	895	−.07^*^	−.17^***^	.60^***^	
5. TOFHLA Total Score	888	−.10^**^	−.20^***^	.88^***^	.91^***^

Note. TOFHLA = Test of Functional Health Literacy in Adults.

* *p* < .05;

** *p* < .01;

*** *p* < .001
